# Occurrence and Spatial Distribution of *Dibothriocephalus Latus* (Cestoda: Diphyllobothriidea) in Lake Iseo (Northern Italy): An Update

**DOI:** 10.3390/ijerph17145070

**Published:** 2020-07-14

**Authors:** Vasco Menconi, Paolo Pastorino, Ivana Momo, Davide Mugetti, Maria Cristina Bona, Sara Levetti, Mattia Tomasoni, Elisabetta Pizzul, Giuseppe Ru, Alessandro Dondo, Marino Prearo

**Affiliations:** 1 The Veterinary Medical Research Institute for Piemonte, Liguria and Valle d’Aosta, 10154 Torino, Italy; vasco.menconi@izsto.it (V.M.); davide.mugetti@izsto.it (D.M.); cristina.bona@izsto.it (M.C.B.); sara.levetti@gmail.com (S.L.); mattia.tomasoni@izsto.it (M.T.); giuseppe.ru@izsto.it (G.R.); alessandro.dondo@izsto.it (A.D.); marino.prearo@izsto.it (M.P.); 2 Department of Life Sciences, University of Trieste, 34127 Trieste, Italy; pizzul@units.it; 3 Department of Veterinary Sciences, University of Torino, 10095 Grugliasco, TO, Italy; ivana.momo@edu.unito.it

**Keywords:** food-borne zoonoses, epidemiological survey, diphyllobothriasis, Italy, *Perca fluviatilis*, subalpine lake

## Abstract

*Dibothriocephalus latus* (Linnaeus, 1758) (Cestoda: Diphyllobothriidea; syn. *Diphyllobothrium latum*), is a fish-borne zoonotic parasite responsible for diphyllobothriasis in humans. Although *D. latus* has long been studied, many aspects of its epidemiology and distribution remain unknown. The aim of this study was to investigate the prevalence, mean intensity of infestation, and mean abundance of plerocercoid larvae of *D. latus* in European perch (*Perca fluviatilis*) and its spatial distribution in three commercial fishing areas in Lake Iseo (Northern Italy). A total of 598 specimens of *P. fluviatilis* were caught in 2019. The total prevalence of *D. latus* was 6.5%. However, there were significant differences between areas (10.2% North; 7.3% Center; 1.5% South) (Chi-square test, *p* = 0.0018). The mean intensity of infestation ranged from 1 larva in southern area to 1.2 larvae in both the central and northern (Pisogne) areas. In addition, the mean abundance ranged from 0.02 in the southern area to 0.26 in the northern area (Pisogne). The total number of larvae (anterior dorsal—AD = 21; anterior ventral—AV = 1; posterior dorsal—PD = 15; posterior ventral—PV = 5) differed significantly between the four anatomical quadrants (Kruskal–Wallis test; *p* = 0.0001). The prevalence of *D. latus* plerocercoid larvae in European perch from Lake Iseo has long been investigated, but without an appropriate sampling design. With the present study, a broader analysis in spatial distribution has been added to the existing literature, revealing new information about *D. latus* distribution and occurrence in Lake Iseo, with new data that will be useful for health authorities and future studies.

## 1. Introduction

*Dibothriocephalus latus* (Linnaeus, 1758) (Cestoda: Diphyllobothriidea; syn. *Diphyllobothrium latum*) is one of the most frequent agents of diphyllobothriasis in humans [1]. *D. latus*, commonly known as the broad tapeworm or fish tapeworm, can be found in the subarctic and temperate areas of the Eurasian Continent; it is occasionally reported in the Arctic and Australia [2]. *D. latus* has a complex life cycle: two intermediate hosts (crustaceans and fish), and definitive hosts (fish-eating mammals including humans) [2].

Adults of *D. latus* living in mammalian hosts produce eggs, which exit the host with faeces. The eggs are typically washed into freshwater lakes where they are eaten by a copepod [2]. The coracidium larva, which hatches from the egg, sheds its epithelium and further develops into the procercoid inside a copepod, the first intermediate host. Transformation into the fully infective procercoid takes several weeks to be completed [2]. From there, the procercoid transfers hosts to a fish (i.e., *Perca fluviatilis*) via ingestion of the copepod intermediate host. There, it migrates to the flesh of the fish and further develops into the plerocercoid. The plerocercoid may pass through other paratenic hosts (i.e., larger *P. fluviatilis*), until finally consumed by a mammalian (i.e., dogs, humans) definitive host [1,2].

The host specificity of *Dibothriocephalus* species is quite broad, while humans may be the main definitive host, especially for *D. latus* [3]. In Europe, the European perch (*Perca fluviatilis*) is the most common host for *D. latus*, the Northern pike (*Esox lucius*) acts as a paratenic host, and the burbot (*Lota lota*) plays a minor role in human diphyllobothriasis [4]. *P. fluviatilis* has a wide distribution throughout the Northern Hemisphere and it is a suitable host for several endohelminth and zoonotic parasites [4,5,6,7,8]. The European perch is a commercially important fish, especially for local fisheries; it is often used in raw and cooked traditional dishes prepared at home and in restaurants [8].

Diphyllobothriasis is caused by the consumption of raw, undercooked, or improperly processed fish products (salted or marinated fish fillets) harboring infective plerocercoid larvae [9,10]. Symptoms involve vitamin B12 deficiency in 40% of cases and gastrointestinal signs, such as abdominal pain and diarrhea in 20% [11]. Weight loss results from parasite nutrient absorption [12]. Despite the worm’s large size and mechanical effect on the definitive host, infections are generally mild or asymptomatic and lead to underestimation of the prevalence of diphyllobothriasis in humans [13,14]. Available data on this fish-borne parasitic zoonosis report that 20 million people are infected worldwide [15].

*D. latus* is endemic in the subalpine lakes of Italy, Switzerland, and France [16]. At the beginning of the past century, diphyllobothriasis was widespread in its endemic area, but it apparently disappeared since the early 1980s [4]. Starting in 2000, it has re-emerged, particularly in the shores of the large subalpine lakes [4,16,17,18,19]. The increasing popularity of cold smoked, marinated or raw fish products seems to be the main reason for the return of diphyllobothriasis [3,15,20,21]. Furthermore, advanced logistics now allow the long-distance trade of unfrozen fish products from around the world, and plerocercoid larvae can resist for days on fish that are merely chilled [22]. Moreover, allochthonous *Diphyllobothrium* infections (*D. dendriticum* and *D. nihonkaiense*) in Europe have been associated with the importation of unfrozen fish products or fish consumed abroad [23,24,25,26,27,28]. Furthermore, parasitic infestations in fish products appear repugnant to consumers and may have negative effects on its commercial value [29].

Health authorities are aware that fish-borne parasitic diseases reduce the commercial value of the products and also affect the economy of the seafood trade. In response to these issues, health authorities have enacted measures to reduce food-borne illness [30,31,32]. Although *D. latus* has long been studied, many aspects of its biology, epidemiology, and distribution remain patchy. The aim of this study was to investigate the prevalence and the spatial distribution of *D. latus* in *P. fluviatilis* from three main commercial fishing areas in Lake Iseo, a subalpine lake in Northern Italy (Lombardy) with recreational tourism and fishery activities. The findings fill our knowledge gaps in *D. latus* epidemiology and may be useful for health authorities and future research. To obtain accurate information on the presence of parasites in an area, appropriate sampling methods, coupled with validated diagnostic techniques, should be applied.

## 2. Materials and Methods

### 2.1. Fish Sampling and Inspection

**Figure 1:**
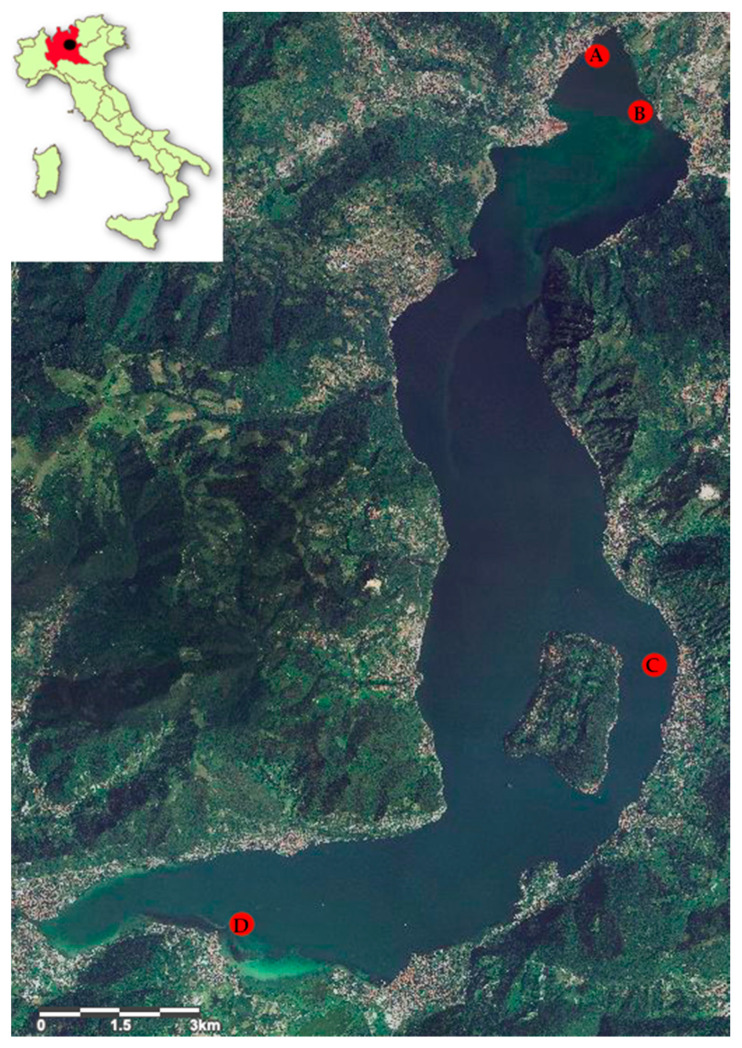
Lake Iseo (Lombardy, Northern Italy) and location of sampling sites (in red). **A**: Costa Volpino (northern area, 45°49′01.2″ N 10°05′18.1″ E); **B**: Pisogne (northern area, 45°48′26.8″ N 10°04′23.0″ E); **C**: Carzano (central area, 45°42′48.3″ N 10°05′48.5″ E); **D**: Clusane (southern area, 45°39′45.3″ N 10°00′46.2″ E).

**Figure 2:**
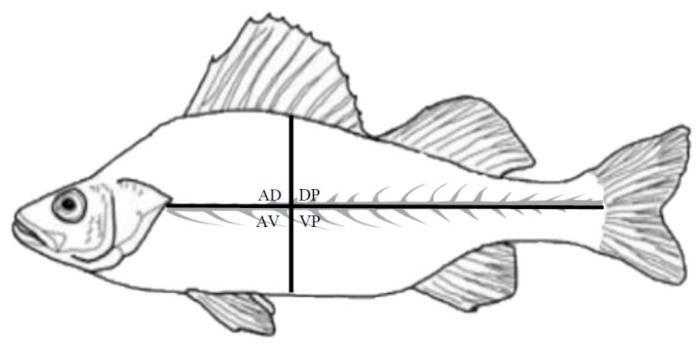
Number and site by quadrant of each larva recorded in the musculature: anterior ventral (AV), anterior dorsal (AD), posterior ventral (VP), and posterior dorsal (DP) quadrants.

In 2019, a parasitological survey was carried out on *Perca fluviatilis* in three areas of Lake Iseo (northern Italy) (Figure 1). Each sampling site was georeferenced and selected to cover the largest surface area of the lake devoted to commercial fishing. A total of 205 fishes were sampled from Clusane (southern area, 45°39′45.3″ N 10°00′46.2″ E), 137 from Carzano (central area, 45°42′48.3″ N 10°05′48.5″ E), and 256 from the northern area where the fishes were caught at two different sites: 233 specimens from Costa Volpino (45°49′01.2″ N 10°05′18.1″ E) and 23 from Pisogne (45°48′26.8″ N 10°04′23.0″ E). The lake depth in the fishing zones ranged from 0 to 110 m. *P. fluviatilis* was caught at a maximum depth of 20 m at each sampling site. The fish were caught by commercial fishermen using mesopelagic gill nets (mesh size 2.5 cm) and kept refrigerated (4 °C), until arrival at the Fish Diseases Laboratory, Istituto Zooprofilattico Sperimentale del Piemonte, Liguria e Valle d’Aosta (Turin, Italy). The fish were measured for total length (cm) and total weight (g), sexed, and then subjected to parasitological examination of musculature and visceral organs (earth, liver, digestive tract, swim bladder, gonads, and spleen) to detect plerocercoid larvae. Each filet was cut in thin slices (approximately 5 mm) and carefully inspected using a white light transilluminator (UVP White Light Transilluminators, TW-43, Analytik Jena, Jena, Germany). Parasites were isolated using dissecting needles, counted, and fixed in 70% ethanol. Morphological identification of the plerocercoid larvae was performed according to Andersen and Gibson [33]. Larvae were placed in 0.9% NaCl solution and then fixed in 96% molecular-grade ethanol for molecular identification (ethanol solution 96%, molecular biology grade, regulated, Fisher BioReagents™, Fisher Scientific, Pittsburg, PA, USA). The site by quadrant of each larva in the musculature was recorded: anterior ventral (AV), which is the belly flap, anterior dorsal (AD), posterior ventral (VP), and posterior dorsal (DP) (Figure 2).

### 2.2. Molecular Analysis

The whole-body of each retrieved parasite was subjected to DNA extraction on silica columns using a commercial kit (Extractme Genomic DNA kit, Blirt S.A., Gdańsk, Poland). DNA was extracted according to the manufacturer’s instructions. The extracted DNA underwent multiplex PCR targeting the cytochrome c oxidase subunit 1 (*cox1*) gene of 4 parasites of the genus *Dibothriocephalus* (*D. latus*, *D. dendriticum*, *D. pacificum*, *D. nihonkaiense*) by means of the method described in Wicht et al. [34]. PCRs were conducted on a 2720 Thermal Cycler 132 (Applied Biosystems, Foster City, CA, USA). Reactions were carried out in a volume of 25 μL, including 12.5 μL of Taq™ DNA Polymerase (TaKaRa Bio Europe SAS, Saint-Germain-en-Laye, France), 0.3 μM of each primer, and 5 μL of parasites DNA. The thermal protocol remained unchanged with respect to the reference paper [34]. A positive control (synthetic plasmid containing *cox1* gene sequence of *D. latus*), a negative extraction control, and a negative PCR control (Nuclease-Free Water) were used for each reaction. The PCR products were visualized by electrophoresis on 2% agarose gel and subsequently purified directly from agarose by Extractme DNA Gel-Out kit (Blirt S.A., Gdańsk, Poland). The purified amplicons were subjected to sequencing according with the Sanger methodology; the primers MulLat3 (5′-GGGGTGTTACGGGTATTATACTC) and MulDenCom (5′-ATGATAAGGGAYAGGRGCYCA) described by Wicht et al. [34] were used. The obtained sequences were assembled with MEGA X and subjected to identification by NCBI Nucleotide BLAST.

### 2.3. Statistical Analysis

The Shapiro–Wilk test was used to verify the normality distribution of the data. The prevalence of infestation was calculated for each area (including the two sites in the northern area) and the entire lake. Differences in the prevalence of infestation between the sampling areas were tested using the Chi-square test. Prevalence, mean intensity and mean abundance of infestation were calculated according to Bush et al. [35]. The 95% confidence intervals (95% CI) were also presented for prevalence values. The non-parametric Kruskal–Wallis test was used to verify the differences in the site of larvae infestation in the musculature (AD, AV, DP, VP) and to check differences in biometric parameters (total length and weight) between fish (both male and female) captured from the sampling sites.

Dunn’s post hoc test was used for multiple comparisons. Significance was set at 0.05%. Spearman’s rank correlation coefficient (ρS) was used to test for correlations between biometric characteristics (total length and total weight) and sex and presence of plerocercoid larvae. Statistical analysis was performed using GraphPad Prism version 8.0.2 software (GraphPad Software, San Diego, CA, USA)

## 3. Results

A total of 598 specimens of *P. fluviatilis* were caught between February 2019 and December 2019 (Table 1) and examined for the presence of *D. latus*. Kruskal–Wallis test did not show any differences in total length and weight between the fish from the selected sampling sites. No visible lesions in external and internal organs were observed. The prevalence of plerocercoid larvae of *D. latus* was: 10.2% (26 out of 256) (95% confidence interval (CI) 7–14.5) in the northern area; 7.3% (10 out of 137) (95% CI 4–12.9) in the central area; and 1.5% (3 out of 205) (95% CI 0.4–4.2) in the southern area. For the two sampling sites in the northern area, the prevalence was 9% (21 out of 233) (95% CI 6–13.4) and 21.7% (5 out of 23) (95% CI 9.7–41.9) for Costa Volpino and Pisogne, respectively. The chi-square test showed significant differences in the prevalence between the three areas (χ^2^ = 14.29; *p* = 0.0018). The total prevalence was 6.5% (39 positives out of 598) (95% CI 4.8–8.8).

Table 2 presents the mean intensity and the mean abundance of infestation for each area. The total number of larvae (AD = 21; AV = 1; DP = 15; VP = 5) differed significantly between the four anatomical quadrants (Kruskal–Wallis test; *p* = 0.0001), with significant differences between AD and AV (Dunn test; *p* = 0.0003), AD and VP (Dunn test; *p* = 0.014), and DP and AV (Dunn test; *p* = 0.014). Spearman’s rank correlation did not show any correlations between biometric characteristics and sex and the presence of plerocercoid larvae.

**Figure 3:**
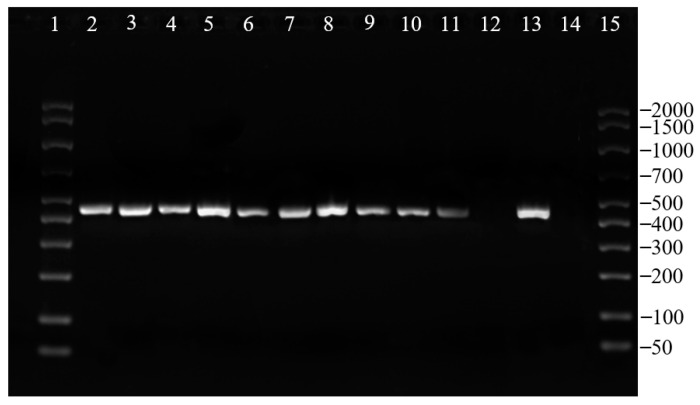
Results of the PCR performed on part of the found parasites. Lane 1 and 15: molecular weight marker (AmpliSize Molecular Ruler 50–2000 bp, Bio-Rad, Italy). Lane 2 to 11: DNA of parasites analyzed in the study. Lane 12: negative extraction control. Lane 13: positive PCR control. Lane 14: negative PCR control.

Biomolecular analysis by multiplex PCR allowed to identify all plerocercoid larvae as *D. latus* (Figure 3). Gene sequencing confirmed the results obtained by PCR, allowing also to highlight a 100% homology with previously published parasite sequences. The portion of *cox1* gene sequenced of all the parasites of the study revealed the same sequence, then deposited in the GenBank (accession number: MT479180).

## 4. Discussion

The total prevalence (6.5%) that we recorded is in line with the 7.6% reported by Gustinelli et al. [4]. Previous studies reported higher prevalence: 25% [36,37,38], 15.7% [39], and 12.8–22.8% [1]. Published historical data show wide variation in prevalence, which could result in a misleading evaluation of the occurrence of *D. latus* in Lake Iseo. In addition, the studies reported only the total prevalence, without georeferencing the sampling sites. We found a significant difference in prevalence between the three sampling areas. The spatial distribution of *D. latus* can also give us information about its life cycle. Ineffective sewage treatment systems are known to permit the contamination of lakes with *D. latus* eggs released by infected humans [4,5,6,7,8,9,10,11,12,13,14,15,16,17,18,19,20,21,22,23,24,25,26,27,28,29,30,31,32,33,34,35,36,37,38,39,40]. In fact, fecal pollution plays a key role in maintaining the parasite’s life cycle and the persistence of diphyllobothriasis in the subalpine area [3,4,41]. The reproductive potential of *D. latus* is very high (up to 1 million eggs is produced each day) [40,42], and even sporadic human cases can give rise to a high prevalence of pleroceroid larvae in a fish population [42]. In light of these considerations and the data obtained, we assume that the northern sampling area is closer to a fecal pollution source than the other sampling sites. This assumption was confirmed by the water quality data from Legambiente monitoring campaigns [43], which revealed higher fecal contamination in the northern and central areas of the lake compared to the southern area.

The spatial distribution of *D. latus* can also be associated with the biological characteristics of the intermediate host. European perch show remarkable site fidelity to the littoral zone and strong homing ability [44]. These tendencies may be related to the patchy distribution and abundance of their preferred prey [45]. Moreover, perch ecology could be linked to the difference in prevalence between the three fishing zones. Plerocercoid larvae were found only in the fillets and the intensity of infestation was in line with published data [1,46]. Furthermore, plerocercoid larvae were chiefly detected in the anterior dorsal (AD) portion, in accordance with Prearo et al. [21]. In European perch, the migration of the larvae probably occurs mainly at the level of the first part of the digestive tract, with subsequent development and localization in the AD portion. At this site, the muscular masses are particularly developed, with a higher success of transmission through the fish consumption [21].

The intensity of infestation of plerocercoid larvae in *P. fluviatilis* seems to imply a high success rate of infection and survival ability when accidentally ingested by the definitive host [4]. No plerocercoid larvae were found in the visceral cavity, which is due to the size of the fish caught for this study. In fact, larger perch prey on smaller infected perch, acting as a paratenic host, as occurs in burbot or in pike [1].

In Lake Iseo, crustacean populations are predominantly represented by Copepoda, the calanoid *Copidodiaptomus steueri* and the cyclopoids *Mesocyclops leuckarti* and *Cyclops abyssorum* [47]. These species are known to be suitable hosts for *D. latus* [48]. Because data on the occurrence of procercoid larvae in copepods are still scarce, further studies are needed to fill this knowledge gap in the *D. latus* life cycle. Environmental monitoring of wastewater, food safety, and molecular epidemiology are key to managing the risks associated with this parasite. The persistence of this parasite in subalpine ecosystems involves many biotic and abiotic factors that are arduous to evaluate individually; a holistic approach is therefore needed. For this reason, we believe that a single prevalence value could misrepresent the complexity of the lake’s ecosystem.

## 5. Conclusions

The prevalence of *D. latus* plerocercoid larvae in European perch from Lake Iseo has long been investigated, but without an appropriate sampling design. With the present study, a broader analysis in spatial distribution has been added to the existing literature, improving the data available for health authorities. Furthermore, our sampling design may be advantageously applied to study the presence of this parasite in other aquatic species or lentic ecosystems.

Zoonotic diseases, especially parasitic infections, are dependent on the abundance of intermediate hosts, and environmental ecological and climatic conditions are key factors in their persistence [49]. Many aspects of *D. latus* biology and ecology remain unknown; therefore, new knowledge is needed. A multidisciplinary approach could provide a better way to fill the current knowledge gaps. Parasitological knowledge needs improvement and should be part of public health planning that integrates field-based research, risk assessment, and adoption of predictive spatial epidemiological models. Public health education, food safety control, and consumer education on the risks of raw fish consumption are fundamental to control and prevent diphyllobothriasis.

## Figures and Tables

**Table 1:** Area, sampling site, total length (Lt), total weight (W), and sex of fish (N) sampled in Lake Iseo (ND = sex not determined).

Area	Site		Female		Male		ND		N
			Lt (cm)	W (g)	Lt (cm)	W (g)	Lt (cm)	W (g)	
North	Costa Volpino	Mean	17.8	71.5	17.7	68.7			
		Median	17.5	64	17.5	65			
		Standard deviation	1.6	22.6	1.7	17.7			
		Max	25	196	24	168			
		Min	14	43.4	9	9.1			
		No.	124		109		0		**233**
	Pisogne	Mean	16.2	60.4	16	56.2			
		Median	16.2	62.2	16	55.7			
		Standard Deviation	0.6	5.9	3.1	20.3			
		Max	17	66.7	18	84.3			
		Min	15.5	51.6	9	9.1			
		No.	6		17		0		**23**
Center	Carzano	Mean					17.3	64.4	
		Median					17	60	
		Standard Deviation					2.9	31.6	
		Max					25	211	
		Min					9	2	
		No.	0		0		137		**137**
South	Clusane	Mean	17	74	16.8	71.6	18.7	95.9	
		Median	16.5	65	16.5	64.4			
		Standard Deviation	2.2	31.8	2.2	37.7	52.2	3.9	
		Max	32	330	26.5	265			
		Min	11.5	17	13.5	28			
		No.	167		36		2		**205**

**Table 2:** Sampling area, prevalence (%), mean intensity, and mean abundance of plerocercoid larvae.

Sampling Area	Prevalence (%) (95% Confidence Interval)	Mean Intensity	Mean Abundance
North—Costa Volpino	9 (6–13.4)	1.14	0.10
North—Pisogne	21.7 (9.7–41.9)	1.2	0.26
Center	7.3 (4–12.9)	1.2	0.09
South	1.5 (0.4–4.2)	1	0.02
